# Cleavable PEGylation: a strategy for overcoming the “PEG dilemma” in efficient drug delivery

**DOI:** 10.1080/10717544.2017.1388451

**Published:** 2017-10-25

**Authors:** Yan Fang, Jianxiu Xue, Shan Gao, Anqi Lu, Dongjuan Yang, Hong Jiang, Yang He, Kai Shi

**Affiliations:** Department of Pharmaceutics, School of Pharmaceutical Science, Shenyang Pharmaceutical University, Shenyang, China

**Keywords:** PEGylation, cleavable PEG, drug delivery, nanocarriers, strategy

## Abstract

To prolong the circulation time of drug, PEGylation has been widely used via the enhanced permeability and retention (EPR) effect, thereby providing new hope for better treatment. However, PEGylation also brings the "PEG dilemma", which is difficult for the cellular absorption of drugs and subsequent endosomal escape. As a result, the activity of drugs is inevitably lost after PEG modification. To achieve successful drug delivery for effective treatment, the crucial issue associated with the use of PEG-lipids, that is, “PEG dilemma” must be addressed. In this paper, we introduced the development and application of nanocarriers with cleavable PEGylation, and discussed various strategies for overcoming the PEG dilemma. Compared to the traditional ones, the vehicle systems with different environmental-sensitive PEG-lipids were discussed, which cleavage can be achieved in response to the intracellular as well as the tumor microenvironment. This smart cleavable PEGylation provides us an efficient strategy to overcome “PEG dilemma”, thereby may be a good candidate for the cancer treatment in future.

## Introduction

In drug delivery system, the application of nanocarriers which are modified by PEG-lipids has made a great progress (Oberoi et al., 2016). The PEG-lipids have been incorporated to the nanocarriers which include liposomes (Zhang et al., 2016), polymer micelles (Kim et al., 2011), polymer nanoparticles (Li et al., 2012b), solid lipid nanoparticles (Yuan et al., 2013), and so on. The PEG-lipids can prolong the circulation time of drugs *in vivo* and promote their permeability and function (Hatakeyama et al., 2007; Maeda, 2012). However, a lot of new problems are brought after the PEGylation (Hatakeyama et al., 2013), which not only affect the interaction between nanocarriers and drugs, but also influence the interaction between nanocarries and cells (Bian et al., 2010). Therefore, how to solve these problems is highly on demand. Based on the mentioned problems, this paper introduces corresponding strategies to minimize the PEGylation dilemma while ensuring the advantages of the PEGylated nanocarriers.

The new cleavable PEG derivatives are characterized as environmentally sensitive, which bonds are easy to break under physiological and pathological conditions. They can not only extend the cycle time of drugs, but also ensure that the PEG detached from the surface of nanocarriers in the target position so as to facilitate the penetration of drugs into cells. This paper summarizes various PEG derivatives with cleavable bonds for overcoming PEGylation dilemma, including peptide bonds (Kulkarni et al., 2014; Lin et al., 2015), disulfide keys (Yan et al., 2014; Wu & Yan, 2015), vinyl ether bonds, hydrazone bonds (Kelly et al., 2016), and ester bonds (Xu et al., 2008). The cleavage of PEG derivatives from carriers was achieved in response to the extracellular as well as intracellular environment, which facilitates the cellular uptake and endosomal escape.

## PEGylation dilemma

### Challenges in development of PEGylated nanocarriers

With the application of PEG-lipids in the nanocarriers, PEGylation dilemma has brought a serious challenge to the development of PEGylated nanocarriers. After PEGylation, nanocarriers cannot be effectively receptor-mediated endocytosis in cancer cells of carcinoma tissue (Song et al., 2002; Mishra et al., 2004; Magarkar et al., 2012). PEGylation inevitably brings the following problems. First, the steric hindrance of PEG chains hinders target cells to uptake drugs (Vance & Marr, 2015). To prevent the recognition of opsonins and subsequent phagocytosis by reticuloendothelial system (RES), a dense hydrophilic PEG shielding are often needed. But these long polymer chains may block the targeting ligands of nanocarriers from binding to the corresponding receptors on cell surface (Hatakeyama et al., 2013). Moreover, the PEG layer may also interfere with the release of drugs from the vehicles (Sanchez et al., 2017).

Second, PEGylation strongly hinders endosomal escape of nano-vehicles, leading to significant loss of activity of the delivery system (Tagalakis et al., 2014). It is well known that endocytosis is the major route for the cellular transport of nanomedicine (Shete et al., 2014). Upon clathrin-mediated internalization at the plasma membrane, the endocytosed cargos are first delivered into early endosomes, where the internal pH value is around 5.0-6.5. Then the early endosomes mature into late endosomes that subsequently fuse with intracellular organelles called lysosomes, which lumen's pH value (4.5–5.0) is optimal for the enzymes involved in hydrolysis. Thus, a limiting step in achieving an effective delivery is to facilitate the endosomal escape and ensure cytosolic delivery of the therapeutics (Paliwal et al., 2015).

Third, accelerated blood clearance (ABC) phenomenon will be produced *in* after repeated injections of PEGylated liposomes (Xu et al., 2014, Kierstead et al., 2015, Wang et al., 2015). Both anti-PEG immunoglobulin M and complement system can trigger the ABC phenomenon (Li et al., 2012a, Shimizu et al., 2015). This is an unexpected pharmacokinetic change resulted from a second dose of conventional PEGylated liposomes. Moreover, other nanocarriers with PEGylation have also been found with this phenomenon after treated by the second dose. With this phenomenon, repeatedly administered PEGylated nanocarriers would be rapidly cleared from systemic circulation due to the accelerated accumulation in the macrophage system (Lila et al., 2013). Among the possible considerations to alleviate the induction of the phenomenon, such as changing the administration regimen (Saadati et al., 2013), reducing the density of PEG on liposome surface and using alternative polymers (Ishihara et al., 2010; Wang et al., 2017), cleavable PEGylation provide us a promising alternative. These cleavable PEG-lipid derivatives could lessen or eliminate the ABC phenomenon produced by repeated injection of PEGylated liposomes or vesicles (Xu et al., 2010; Chen et al., 2011). In addition, the factors that influence vesicles internalization involve non-cleavable chemical bond (He et al., 2014; Zeng et al., 2014), conformation cloud (Yoshino et al., 2012), and hydration film (Basile et al., 2012) . The presence of PEG suppressed the fusion between liposomes and the cellular and endosomal membranes (Felber et al., 2012). These are a series of negative effects known as the “PEG predicament” or “PEG dilemma” (Wei et al., 2012). Therefore, a successful drug delivery system for effective treatment requires a rational strategy and the design of carrier systems to overcome the issues associated with the use of PEG-lipids. Based on the dilemma, cleavable PEG derivatives will be summarized in this paper.

### Traditional PEG-lipids-modified nanocarriers

It is well known that conventional nanocarriers without surface modification are very unstable in plasma and fail to effectively deliver their contents to the target tissues or cells. Therefore, improving their stability has become a key issue. (Lankveld et al., 2011). In order to obtain a longer cycle time *in vivo*, PEG derivatives are used as the coating layer of nanomaterials to increase their surface hydrophilicity and steric hindrance, thereby extending their circulation time *in vivo*. It has attracted attention of many researchers (Wang et al., 2010a; Wang et al., 2012). The chemical bonds between conventional PEG-lipids are usually amide (Zhang et al., 2004) or ether bonds (He et al., 2014), which have high chemical stability and thus are difficult to be removed from the carriers. The steric effect of PEG-lipids would be resistant to degradation of the PEGylated nanocarriers *in vivo*, preventing interactions between nanocarriers and target cells (Maeda & Fujimoto, 2006). Therefore, the traditional PEG-lipid materials can hinder cellular uptake and subsequent endosomal escape of the drug (Romberg et al., 2008). To solve the PEG dilemma, this article presents various solutions for the traditional PEG-lipids.

### Discrepancies between non-cleavable and cleavable PEG-lipids

Both traditional and cleavable PEG-lipids-modified carriers can extend the drug circulation time *in vivo*. In general, the non-cleavable PEG-lipids are incorporated to carriers by covalently linked via amide or ether bonds, which are insensitive to the environment (Maeda & Fujimoto, 2006). To achieve more efficient drug delivery efficiency, new PEG-lipid derivatives are used to bind with carriers and release drugs under certain conditions (Mercadal et al., 2000; Ghigo et al., 2006; Hatakeyama et al., 2009). The use of cleavable carriers is expected to increase the cycle time and improve drug absorption *in vivo*. But because of the steric effect of PEG-lipids, the non-cleavable PEG-lipids will hinder the drug from cellular uptake and subsequent endosomal escape (Romberg et al., 2008). In order to overcome these shortcomings, nanocarriers with cleavable bonds are put forward and studied by many researchers. Upon stimulation from the intracellular and extracellular microenvironment of cancer cells, the PEG-lipids can fall off from the surface of carriers. There are structural differences between these two types of nanocarriers, mainly in the aspect of non-cleavable bonds (traditional nanocarriers) and cleavable bonds (smart nanocarries). The difference of effect is that the traditional PEGylation brings “PEG dilemma”, while the cleavable PEGylation can not only prolong the circulation time of drugs, but also increase their absorption. Based on these differences between structures and efficacy, studies have shown that the key is to enable carriers cleavable.

## Strategies for overcoming the “PEG Dilemma”

### Action mechanisms of cleavable PEG-lipids

**Figure 1.**
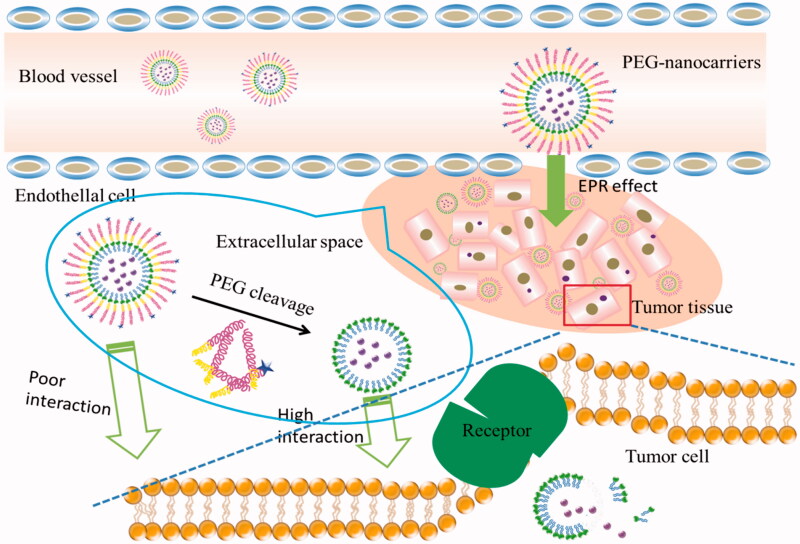
The bonds are cleaved in the target site.

To overcome PEGylation dilemma, various carriers with PEG moiety cleaving systems have been developed that release drugs when they were exposed to the appropriate stimulus at the target site. Once stimulated appropriately, cleavable PEG-lipids can escape from the surface of the nanocomposite and the loaded drugs can be taken up by the cancer cells. Most of the cleavable PEG derivatives have been designed to be cleaved in response to the extracellular or intracellular microenvironment, such as temperature, pH, specific enzyme, reductive conditions, and so on. For example, pH-sensitive liposomes can release drugs by the breaking of hydrazone bonds in the acidic environment of tumor tissues (Sawant et al., 2006; Dong et al., 2013). Liposomes containing the peptide bonds achieve the desired effects after bonds cleavage under the action of matrix metalloproteinases (MMPs) in the body (Koutroumanis et al., 2013; Wang et al., 2014). PEG derivatives conjugated with disulfide bonds can be cleaved in the cell by the glutathione-mediated reduction environment via thiol-disulfide exchange reactions (Wang et al., 2014). In addition, carries with vinyl ether bonds and ester bonds can also be cleaved under acidic or oxidative conditions (Terada et al., 2006). These cleavable PEG-lipids have corresponding fracture mechanisms, so the application of them can achieve long-term circulation and promote drug absorption at a specific location. The cleavable mechanisms (taking solid lipid nanoparticle as an example) are shown in the Figure 1.

### Vinyl ether bond and its application

Because the vinyl ether bond must be non-oxidative and either neutral or basic, the bond is labile under acidic or oxidative conditions. Shin et al. synthesized four structurally related acid-labile PEG-lipids, which are linked via vinyl ether bond (Shin et al., 2003). Acid-catalyzed hydrolysis of the vinyl ether bond destabilized liposomes by removal of the sterically stabilizing PEG layer, thereby promoting contents release on the hours timescale at pH <5. To mitigate the limitations of PEG-modified carriers, a light triggered liposome was developed which was modified by cholesterol derivatives via a cleavable vinyl ether linkage so that the PEGylated coatings can be efficiently removed by photosensitiser-generated reactive singlet oxygen. After the cleavage of the PEG moiety, the intracellular uptake of the liposome improved significantly (Komeda et al., 2013). Shin et al. synthetized four structurally related materials, which are composed of acid-labile PEG-conjugated vinyl ether lipids and used to stabilize dioleoylphosphatidyl ethanolamine (DOPE) liposomes. Acid-catalyzed hydrolysis of the vinyl ether bond destabilized these liposomes by removal of the sterically stabilizing PEG layer, thereby promoting contents release on the hours timescale at pH <5. Structure-property correlations of these compounds suggested that single vinyl ether linkages between the PEG headgroup and the lipid backbone produce faster leakage rates than others (Shin et al., 2003).

### Hydrazone bond and its application

**Figure 2.**
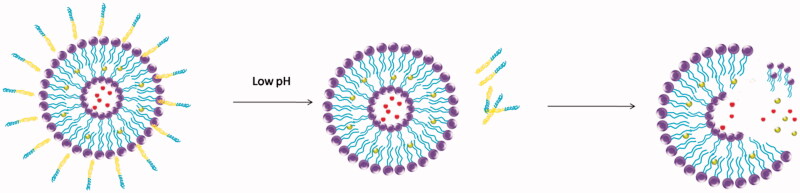
The pH-sensitive liposome follows the change of pH value, which including hydrazone bond and vinyl ether bond.

When using the PEG-lipid nanocomposites, the issue of how to achieve rapid and effective release of the drugs is very important. In addition to the vinyl ether bond, hydrazone bonds are other typical acid-labile ones. This linker was first used to couple monoclonal antibody and chemotherapeutic drugs to the PEG chains (Hansen et al., 1995; Rodrigues et al., 1999). The resulted acid-sensitive PEG conjugates showed improved activity against cancer cells *in vitro*. Their triggering mechanism in acid environment, as shown in Figure 2, is well correlated with the extracellular pH of cancer tissue. Furthermore, it is shown that the application of acid-sensitive liposomes in the treatment of tumor is reasonable and effective (Lee et al., 2005).

**Figure 3.**
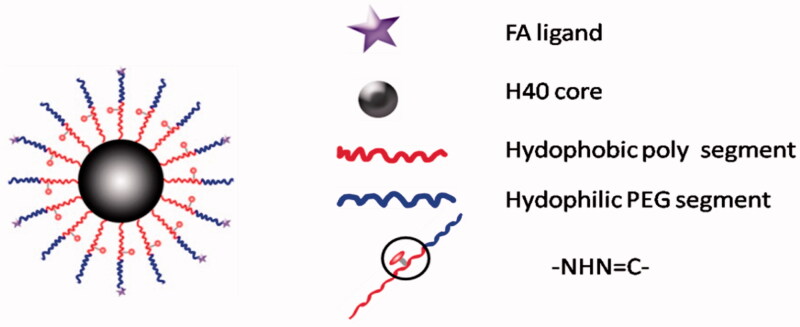
Schematic structure of the H40-P (LA-DOX)-b-PEG-OH/FA copolymer.

It is well known that doxorubicin (DOX) is widely used in the treatment of a wide variety of tumors, including hematological malignancies, many types of carcinoma, and soft tissue sarcomas (Danquah et al., 2011). However, high toxicity, short half-life, poor water solubility, and multi-drug resistance limit its therapeutic efficacy (Hu et al., 2009; Cuong et al., 2010). Therefore, a novel cleavable micelles, which structure is shown in Figure 3, was developed (Prabaharan et al., 2009; Jiang et al., 2013). In this study, DOX was covalently conjugated onto the hydrophobic segments of the amphiphilic block copolymer via a hydra pH-sensitive hydrazone bond. The *in vitro* release profiles of the DOX from the micelles showed a strong dependence on the environmental pH values. The increased drug release rate in the acidic medium attributes to the acid-cleavable hydrazone linkage between the DOX and micelles(Bae & Kataoka, 2009). Under acidic conditions (especially pH 5.3), pH-dependent hydrazone bonds are susceptible to hydrolysis and cleavage, which makes it possible to achieve an effective concentration of DOX in a short period of time (Prabaharan et al., 2009). When conjugated to the micellar carriers via pH-sensitive hydrazone linkage along with PEG chain, the anticancer drug of DOX showed a strong dependence on the environmental pH values. (Etrych et al., 2011; Zhou et al., 2011). It was characterized as stable under physiological conditions as well as accelerated releasing in the acidic medium due to the acid-cleavable hydrazone linkage between the DOX and micelles, thereby providing higher cytotoxicity against cancer cells (Patil et al., 2012). These showed that cleavable PEG-modified nanocarriers may be more effective than traditional nanocarriers in tumor therapy.

In another typical example, a multifunctional nanocarrier with PEGylated TATp-modified pH-sensitive liposomes was designed in which pH-labile hydrazone bond was inserted between PEG and PE (PEG-Hz-PE) (Sawant et al., 2006; Kale & Torchilin, 2007b). TAT-p is a kind of cell-penetrating peptide which can pass through the cell membrane directly, and thus make the carriers enter the cancer cells more quickly and effectively. At lower pH environment of hypoxia regions, hydrazone bond is cleaved and exposes the cell penetration functions such as TAT peptide. For example, Kale et al. synthesized a series of acid-sensitive PEG-Hz-PE conjugates having different substituent hydrazone bonds. Their hydrolytic stability under normal and slightly acidic conditions was evaluated. The results demonstrate that the hydrazone bonds derivatized from PEG-Hz-PE-conjugated aromatic aldehydes are very stable in both cases, but the ones from PEG-Hz-PE-conjugated aliphatic aldehydes are easier to hydrolyze (Kale & Torchilin, 2007a). Chen et al. not only studied the pH-dependent degradation of mPEG-Hz-Chol complexes, but also carried out evaluation of their modified pH-sensitive liposomes *in vivo* (Chen et al., 2010). Pharmacokinetic studies suggested that compared with conventional ones, the pH-sensitive liposomes might decrease clearance rate as well as the accumulation toxic effect in liver and spleen. Recently, a new multifunctional immunoliposomal nanocarrier containing pH-sensitive hydrazone bond between the long shielding PEG chains and PE (PEG2k-Hz-PE) has been proposed and studied (Koren et al., 2012). Under normal pH conditions, TATp moieties are shielded by the long PEG chains. Upon the exposure to a lower pH environment, the multifunctional carrier suffered hydrolysis of hydrazone bond and removal of PEG chains, thereby TATp moieties were partially exposed. Enhanced cellular uptake of the TATp-containing immunoliposomes was observed *in vitro* after pretreatment at lowered pH. Furthermore, they showed increased cellular cytotoxicity of B16-F10, HeLa, and MCF-7 cells when pre-incubated at lower pH, indicating TATp exposure and activity. The above mentioned researches indicate that pH-responsive nanocarriers (such as liposomes, nanoparticles, micelles) with hydrazone bond and targeting ligand (folic acid, cell penetrating peptide) will achieve better therapeutic effect in the treatment of tumor.

Composites containing acid-sensitive hydrazone bonds also have other applications. For example, cleavable methoxy polyethylene glycol 2000-hydrazone-cholesteryl hemisuccinate (mPEG-Hz-CHEMS) polymer was designed as a modified pH-sensitive liposome that would selectively degrade under locally acidic vaginal conditions. Under basic or neutral conditions, it can be stable for a long period of time, but suffered cleavage under acidic conditions (pH 5.0) (Chen et al., 2012). For cancer cell imaging, there are also applications of hydrazone bond in constructing pH stimuli-responsive nanoprobe. In the designed PEG-TGA/TGH-capped CdTe quantum dots, the hydrazide on TGH reacts with the aldehyde on PEG and forms a hydrazone bond. At specific pH values, the hydrazone bond ruptured and release the quantum dots, which allows the prepared pH-stimuli-responsive nanoprobes to show fluorescence signals in the imaged cancer cells (Du et al., 2015). Acid-sensitive nanocomposite can also be combined with siRNA to treat cancer, thereby achieving better therapeutic gene silencing. For example, a multifunctional polyethyleneimine (PEI)-Hz-DOX (PHD)/PEI-PEG-Folate (PPF)/siRNA complexes were developed in which DOX was conjugated to PEI via a pH-responsive hydrazone linkage. By the way of acid-triggered manner, that is, hydrazone bond cleavage and endosome/lysosome escape, the complexes release chemotherapy drugs and siRNA against cancerous cells (Dong et al., 2013). Nanocomposite containing hydrazone bond is also used as protective carriers. Peptide aldehydes, such as MG132 (Cbz-leu-leu-leucin) can inhibit proteasome and suppress growth of cancer cells. Nevertheless, they are easily oxidized in the aldehyde functional group and lose their activity *in vivo*. To solve this problem, MG132 was covalently conjugated to a block copolymer composed of PEG and polyaspartate via an acid-labile hydrazone bond (Quader et al., 2014). This bond is stable at physiological condition, but hydrolytically cleavable in the acidic compartments of cell, such as late endosomes and lysosomes. Thus, release of MG132 from protective micelles after the enhanced permeability and retention (EPR) effect-mediated tumor accumulation was facilitated. In addition, the combination of various cleavable bonds such as hydrazone and disulfide bond was employed to design and prepare folate-PEG-coated polymeric lipid vesicles (FPPLVs), where PEG chains and stearyl alcohol moiety were linked on the main chain of dextran by pH-sensitive hydrazone bond and reduction-sensitive disulfide bonds, respectively. This smart pH- and reduction-dual-responsive drug delivery system was found to triggered drug release in response to acidic pH and reducing environments due to the cleavage of hydrazone bonds and disulfide bonds. (Wang et al., 2014). It has also been demonstrated by an *in vitro* cellular uptake study that the FPPLVs lose their PEG coating as well as expose the folate in acidic conditions, which allows them to efficiently enter tumor cells through ligand-receptor interactions. The results show that the combination of the two kinds of bonds has better effect as well as wider application.

### Other pH-sensitive bonds and their application

Amide bond is another kind of acid-labile linkage which is formed between primary amines and unsaturated anhydrides like citraconic anhydride, 2,3-dimethylmaleic anhydride and cis-aconitic anhydride. These bonds suffer cleavage upon exposure to acidic conditions, thereby causing release of the conjugated amine drugs. Sun et al. reported a polymeric nanoparticle based on a bridged PEG and poly(D,L-lactide) (PDLLA) block copolymer for improved cancer therapy. The acid-labile copolymer, denoted as PEG-*Dlink_m_*-PDLLA, was synthesized by covalently connecting PEG and PDLLA segments with an acid-degradable amide bond. Upon arriving at the tumor site, the nanoparticles will lose the PEG layer and increase zeta potential by responding to tumor acidity, which significantly enhances cellular uptake and improves the *in vivo* tumor inhibition rate (Sun et al., 2016). Similarly, schiff bases/imines are formed by the reaction of primary amines and aldehydes/ketones. Zhao et al. designed a new acidly sensitive PEGylated polyethylenimine linked by Schiff base (PEG-s-PEI), which would render pH-sensitive PEGylation nanoassemblies through multiple interactions with indomethacin and docetaxel. At extracellular pH of tumor microenvironment, the nanoassemblies exhibited an excellent performance of acid-induced cleavage, which provided an efficient strategy to target tumor microenvironment (Zhao et al., 2017).

### Ester bond and its application

In view of the fact that ester bonds are susceptible to hydrolysis by esterases that are widespread in the plasma and tissues, a novel cleavable PEG-lipid, that is, mPEG2000-CHEMS (mPEG2000-CHEMS) was developed by Xu et al. (2008). In order to better control the release of the contents, two PEG-lipid derivatives (mPEG-CHEMS and mPEG-CHMC) are linked to the carriers via the ester bonds. In contrast to conventional long circulation materials of mPEG-distearoyl phosphatidylethandamine (DSPE) and mPEG-Chol, the two new conjugates enabled higher degrees of PEG cleavages from modified vesicles. In addition, because of their narrow therapeutic window, the rapid uptake of anticancer agents may induce liver damage. Therefore, in order to achieve better targeting and lower toxicity, as well as assure the continuous interaction of liposomes with the target tissue, a longer blood circulation time may be necessary. The cleavable PEG2000-CHEMS linked via ester bond and galactosylated lipid was used to modify DOX contained liposome (PEG-GalL). Compared with conventional PEGylated liposomes, the PEG-GalL showed unique “sustained targeting” characterized by slowed target releasing DOX and thereby reduced peak concentrations in the liver. Moreover, the inhibitory rate of PEG-GalL DOX to H22 tumors was significantly higher than that of conventional PEGylated liposomes, (Wang et al., 2010b). The esterase cleavable PEG-lipid conjugations are not only applicable to stabilizing vesicles and prolonging their circulation time, but also providing more efficient release of contents by the esterase-catalyzed dePEGylation. The link is via their ester bonds for the vesicle modifications, so the PEG-lipids can be cleaved by esterases. Ester linkages between the PEG and lipid anchors allow the contents to release more rapidly under suitable conditions. PEG cleavage is related to the several factors. On the one hand, PEG cleavage decreased as increase in the molar ratios of the PEG-lipids to vesicles. On the other hand, PEG cleavage gradually increased with the increase of serum concentration. However, related research reported that repeated injection of mPEG-CHEMS liposomes would lead to a minor ABC phenomenon which was accompanied by a slight increase in liver uptake (Dams et al., 2000; Laverman et al., 2001). Studies have shown that the cleavable mPEG-Hz-CHEMS derivatives could lessen or eliminate the ABC phenomenon which is induced by repeated injection of PEGylated liposomes (Chen et al., 2011).

### Peptide bond and its application

**Figure 4.**
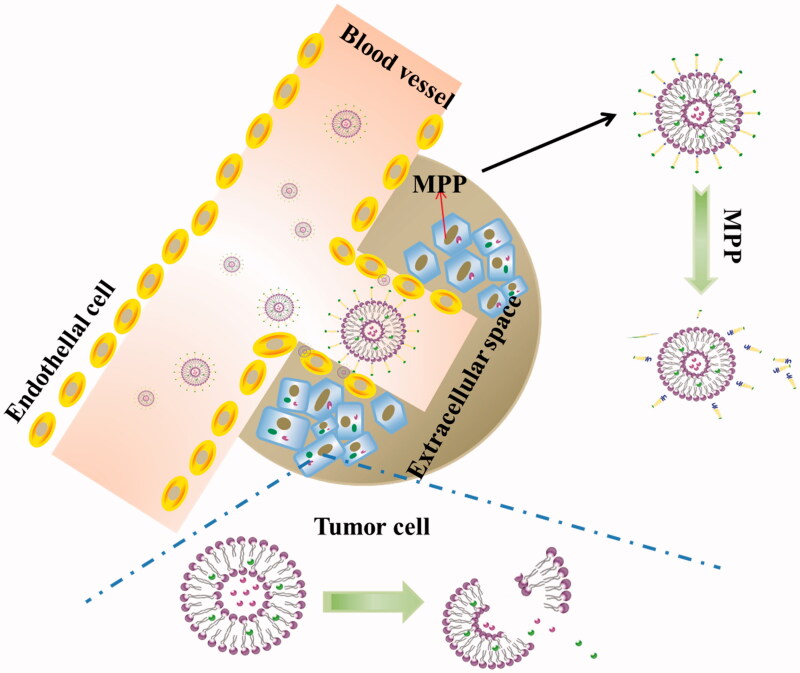
The cleavage mechanism of liposomes containing PPD (PEG-peptide-lipids).

The small peptide fragments used in the connection of PEG and lipid have the advantages of simple synthesis and easy implement. Because these fragments are easily degraded by some endogenous substances in the body, cutting off this chain of PEG segment from carriers can be performed *in vivo.* After surface modification with these peptides-linked PEG, the liposomes cannot only achieve the goal of long cycle *in vivo*, but also increase drug gathering in cancerous tissue. To achieve a tumor-specific cleavable PEG system, the enzymes specifically expressed in a tumor are focused on, such as MMPs. MMPs are highly expressed in tumor cells and distributed in the extracellular space, but their expression levels are low in normal cells (Kim et al., 2001). Normally, the concentration of MMPs in the extracellular supernatant is approximately 20 ng/ml, which is sufficient to trigger PEG cleavage (Hatakeyama et al., 2009). Therefore, the PEG-peptide-lipid conjugates with MMP substrate peptide not only extended the systemic circulation time and promote the accumulation of liposome in tumor cells by EPR effect, but also solved the dilemma of PEG during drug delivery and cellular transfection (Hatakeyama et al., 2007). In order to overcome the limitations of cellular absorption and subsequent endosomal escape of multifunctional envelope-type nano device (MEND) after PEGylation, Hatakeyama et al. developed a PEG-peptide-DOPE (PPD) which can be cleaved in the MMPs rich environment (Hatakeyama et al., 2009). The *in vitro* study revealed that compared to a conventional PEG-modified MEND, PPD modification improved both cellular uptake and endosomal escape. During systemic administration, the optimized PPD-MEND resulted in an approximately 70% silencing activity in tumors as compared to non-treatment (Hatakeyama et al., 2011). The cleavage mechanism of the nanocarriers containing PPD is shown Figure 4.

### Disulfide bond and its application

**Figure 5.**
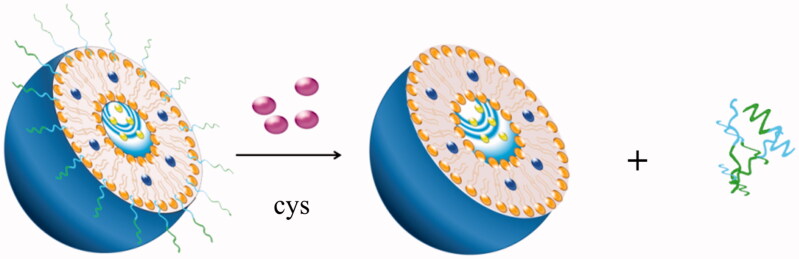
The cleavage mechanism of disulfide bonds-linked PEGylated liposomes.

The PEG shielding layer will lead to significant steric hindrance, which will negatively affect cell uptake and intracellular distribution of nanocarriers. This unexpected effect would impair the biological effectiveness of the encapsulated payload. In order to solve this problem, one of the alternatives is to couple the disulfide bond to PEG, so as to construct a disulfide-bridged cleavable PEGylation. This linkage was first used to bridge PEGylated proteins, by which their advanced structure and biological activity are retained (Shaunak et al., 2006). Due to the fact that intracellular glutathione (GSH) concentration is nearly three orders of magnitude higher than that outside the cell, the disulfide bond can be selectively cleaved in the tumor environment by the significant concentration gradient of GSH, especially in the intracellular region (Dong, et al., 2015). In addition, extracellular low concentration of GSH make the disulfide-linked nanocarriers highly stable before their internalization in the target cells. (Wu et al., 2012). In the early study of disulfide bond, To prepare reduction-triggered liposomes, Oumzil et al. synthesized a PEG detachable nucleotide lipid (DOU-SS-PEG2000) starting from HS-PEG-OMe and uridine. By adding dithiothreitol (DTT) as reducing reagent, the disulfide bond of the PEG chain was cleaved and thus exposed the cationic surface of the liposome (Oumzil et al., 2011). Compared to the common PEGylated ones, DOU-SS-PEG2000-modified liposomes showed improved internalization efficiency in ovarian cancer cells. To prepare targetable sterically stabilized immunoliposomes (SIL) to CD34 + cells, Mercada et al. coupling anti-CD34 My10 mAb to PEG-liposomes containing functionalized PEG-lipid anchor via a cleavable disulfide bond. The disulfide bond was stable in cell culture medium (10% of fetal calf serum) during 8 h and cell-bound SIL can be released from cells by treatment with DTT as reducing agent under mild conditions (Mercadal et al., 2000). For the detachable coating, lipid of dioleoylphosphatidylethanolamine (DOPE) and PEG chain were connected via a disulfide linkage. When adding a reducing agent such as L-cysteine, the thiolytic cleavable spacer (PEG-S-S-DOPE) was cleaved to expose the membrane-permeable ligand (octaarginine) on the liposome surface and thereby internalization of the liposomes was significantly facilitated. (Maeda & Fujimoto, 2006). (seen in Figure 5) Gene transfection efficiency was affected by elective intracellular uptake and sufficient circulation time. It has been found that siRNA is readily degraded by ubiquitous RNases and is not retained *in vivo* for long periods of time (Haupenthal et al., 2006). PEGylation of gene vector has been proven to be one of the most effective ways to prolong *in vivo* circulation time of the genetic payload. In order to meet the special needs of gene transfection, Cai et al. developed PEG-detachable catiomers, which is composed of mPEG-SS-PLL/DNA complexes, as non-viral gene vectors to detach the PEG layers responsive to the intracellular reducing environment (Cai et al., 2011, 2012). *In vivo* study, these complexes showed high transfection efficiency in 293 T and Hela cells under optimized conditions. It will indicate the direction of the clinical application of nonviral gene delivery.

As liposomes are mainly composed of natural chemical components, they can control the retention of drugs, improve the life cycle of blood circulation, and reduce the toxicity of many drugs. Therefore, the liposomal delivery systems have attracted much attentions (Maeda & Fujimoto, 2006; Sawant et al., 2006; Torchilin, 2007; Wang & Thanou, 2010). Recently, researchers have combined the advantages of membrane-permeable ligands (such as octaarginine) with cleavable PEG-lipids (Mei et al., 2014). Adding a reducing agent such as L-cysteine can make the PEG chains rupture and thus releasing R8 (arginine octamer). PEG-modified carriers having a particle size less than 100 nm can cross the endothelial system and accumulate in the tumor, without being RES uptake. When the liposomes are internalized into the cytosol, disulfide bonds can be further cleaved by the cellular glutathione, thereby rupturing liposomes and releasing the drug. In order to promote the intracellular delivery of drugs and genes, new PEG-lipid containing the cleavable coating and the hidden membrane-permeable moieties has been reported., (Maeda and Fujimoto, 2006). Among these carriers, TAT peptide is inserted into the PEG chain which can increase the uptake rate of the cells (Torchilin et al., 2001, 2003; Fretz et al., 2004; Vandenbroucke et al., 2007). The collaborative applications of TAT peptide and PEG *in vitro* and *in vivo* have been confirmed by many literatures for improving delivery capability of liposomes. (Maeda et al., 2004; Kuai et al., 2010; Pappalardo et al., 2009; Torchilin, 2008). For example, incorporation of cleavable PEG5000 into TAT peptide-conjugated stealth liposome (TAT-SL) led to much more tumor accumulation and less liver distribution compared with TAT-SL. In the presence of Cys, the delivery efficiency of TAT-SL enhanced 30% than the SL control. It is a quite promising drug delivery system for cancer diagnosis and treatment in the future (Kuai et al., 2011).

## Conclusions

The “PEG Dilemma” is one of the biggest problems currently plaguing the development of drug delivery systems. At present, there are many ways to solve this problem from various angles. This article summarizes the various applications of the PEG derivatives. The cleavable PEG-lipids overcome the shortcomings of traditional PEGylation and make a big step forward in the treatment of cancer. Several kinds of PEG-lipids are introduced, and the trigger mechanism as well as their applications were briefly introduced. Each cleavable PEG derivative has its own advantages and disadvantages. The mechanism of action *in vivo* needs further study. But from simple assays *in vitro* to study the process of cells, the triggering mechanism has become increasingly clear. Recently, more and more research has focused on the joint cleavable bonds and other ligands. There are also considerable number of reports on combined application of cleavable PEG and cell membrane permeable peptide, which have achieved satisfactory results and thereby broadening the scope of application. Nanocomposites comprising cleavable bonds may become the most effective drug delivery system for cancer treatment in the future. The cleavable PEG-lipids are summarized in Table 1, which may be helpful to the study of the nanocomposites in the future. Comparing the cleavable PEG derivatives listed above, we can draw a conclusion. Most researchers chose pH-sensitive derivatives, which may be related to the microenvironment of cancer cells. In the low pH value of tumor microenvironment, hydrazone bond or vinyl ether bond can be broken more easily. The cleavable PEG-nanocarriers may bring a promising strategy for cancer treatment in future.

**Table 1.** Research of cleavable conjugates-modified DDS.

Trigger mechanism	PEG-lipids conjugate	DDS (drug delivery system)	Cleavable bond	Structure of bonds	Typical structures	Cleavage sites	Reference
Acidolysis (especially pH 5.3)	mPEG-derivatives	Liposome (DOX)	vinyl ether bond	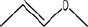	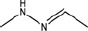	tumor interstitial fluid (pH 6.5-7.2); endosomes (pH 5.0-6.5) and lysosomes (pH 4.5-5.0)	(Shin et al., 2003; Komeda et al., 2013)
	mPEG-b-P(ATMC-co-DTC) mPEG-Hz-CHEMS FPPLVs PEI-PE/pDNA	Liposome (PTX) Micelle (MG132) conjugates (DOX) vaginal delivery gene delivery	hydrazine bond	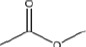	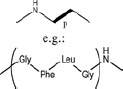		(Kale & Torchilin, 2007b; Prabaharan et al., 2009; Chen et al., 2010; Swati et al., 2011; Chen et al., 2012; Koren et al., 2012; Zhou et al., 2012; Sawant et al., 2012; Dong et al., 2013; Jiang et al., 2013; Koutroumanis et al., 2013; Quader et al., 2014; Wang et al., 2014a; Du et al., 2015)
Hydrolyse (esterase)	mPEG-CHMCS PEG-GalL CL-R8-LP	Liposome (DOX) PEG-GalL DOX	ester bond	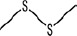	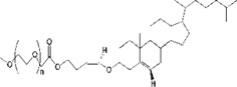	extracellular microenvironment	(Ishida et al., 2001; Wang et al., 2010b; Chen et al., 2011; Hammer et al., 2014; Tang et al., 2014)
Enzymolysis (MPP is expressed highly)	mPEG-GFLG-DSPE mPEG-DTH-DSPA mPEG-DTP-DSPE	Liposome (NOAC) Liposome (DOX) PPp-PTX-SLNs siRNA delivery gene delivery	peptide bond	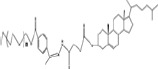	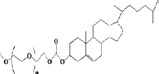	extracellular matrix	(Zhang et al., 2004; Terada et al., 2006; Hatakeyama et al., 2007; Hatakeyama et al., 2009; Zheng et al., 2014)
Thiolysis i) adding dithiothreitol (DTT) *in vitro* ii) a safe cleaving agent L-cysteine (L-Cys) *in vivo*	POPE-SS-PEG5000 PEG-S-S-DOPE mPEG-S-S-DSPE, mPEG-SS-Lysn-r-Hism	Liposome (PEG-S-S-R8) Liposome (My10-SIL) tumor gene therapy siRNA delivery	disulfide bond	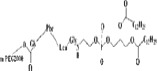	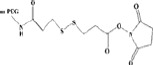	intracellular cytoplasm	(Mercadal et al., 2000; Ishida et al., 2001; Maeda & Fujimoto, 2006; Kuai et al., 2011; Oumzil et al., 2011; Kulkarni et al., 2014; Mei et al., 2014; Zhu et al., 2014)

My10: anti-CD34 My10; mAb: monoclonal antibody; NOAC: N4-octadecyl-1-β-D-arabinofuranosylcytosine; DTH: dithio-3-hexanol; GFLG: Gly-Phe-Leu-Gly-aminoethanol; DTP: dithiodipropi-onateaminoethanol; SLNs: solid lipid nanoparticles; DSPE: distearoyl phosphatidylethandamine; PTX: paclitaxel; SIL: sterically stabilized immunoliposomes; POPE-SS-PEG5000: PEGylated1-palmitoyl-2-oleoyl-snglycero-3-phosphoethanolamine lipid; mPEG-S-S-DSPE: N-[2-w-methoxypoly(ethyleneglycol)-a-aminocarbonylethyl-dithiopropinoyl]-DSPE; DSPE-PEG-TAT: 1,2-distearoyl-sn-gycero-3-phospho-ethanolamine-poly(ethylene glycol)2000 (DSPE-PEG2000)-TAT; DSPE: 1,2-distearoyl-sn-glycero-3-phosphoethanolamine; PEI-PE: polyethyleneimine (PEI) and dioleoylphosphatidylethanolamine (PE); DOPE: dioleoylphosphatidylethanolamine.

## Expectation

Reviewing the above literatures, we can see that PEGylation strategy has undergone the following three steps: simple PEG surface modification, cleavable PEG conjugation, and joint applications of multiple breakable PEG derivatives. In this paper, we introduce the species of PEG-lipid derivatives as well as their triggering mechanism and applications. Cleavable PEGylation is a strategy that can overcome the “PEG dilemma” in efficient drug delivery. With the development of the research, there will be more and more new cleavable PEG derivatives to be developed. At present, many researchers have focused on the combined use of PEG and target ligands. These may bring new ideas for the future and provide more candidates for cancer treatment in the future.

## References

[CIT0001] Bae Y, Kataoka K. (2009). Intelligent polymeric micelles from functional poly(ethylene glycol)-poly(amino acid) block copolymers. Adv Drug Deliv Rev 61:768–84. 19422866 10.1016/j.addr.2009.04.016

[CIT0002] Basile L, Passirani C, Huynh NT, et al. (2012). Serum-stable, long-circulating paclitaxel-loaded colloidal carriers decorated with a new amphiphilic PEG derivative. Int J Pharm 426:231–8. 22306040 10.1016/j.ijpharm.2012.01.038

[CIT0003] Bian X, Shen F, Chen Y, et al. (2010). PEGylation of alpha-momorcharin: synthesis and characterization of novel anti-tumor conjugates with therapeutic potential. Biotechnol Lett 32:883–90. 20238144 10.1007/s10529-010-0242-8

[CIT0005] Cai X, Dong H, Xia W, et al. (2011). Glutathione-mediated shedding of PEG layers based on disulfide-linked catiomers for DNA delivery. J Mater Chem 21:14639–45.

[CIT0006] Cai X, Dong C, Dong H, et al. (2012). Effective gene delivery using stimulus-responsive catiomer designed with redox-sensitive disulfide and acid-labile imine linkers. Biomacromolecules 13:1024–34. 22443494 10.1021/bm2017355

[CIT0007] Chen D, Jiang X, Liu J, et al. (2010). In vivo evaluation of novel pH-sensitive mPEG-Hz-Chol conjugate in liposomes: pharmacokinetics, tissue distribution, efficacy assessment. Artif Cells Blood Substit Immobil Biotechnol 38:136–42. 20337549 10.3109/10731191003685481

[CIT0008] Chen D, Liu W, Shen Y, et al. (2011). Effects of a novel pH-sensitive liposome with cleavable esterase-catalyzed and pH-responsive double smart mPEG lipid derivative on ABC phenomenon. Int J Nanomedicine 6:2053–61. 21976980 10.2147/IJN.S24344 PMC3181064

[CIT0009] Chen D, Sun K, Mu H, et al. (2012). pH and temperature dual-sensitive liposome gel based on novel cleavable mPEG-Hz-CHEMS polymeric vaginal delivery system. Int J Nanomedicine 7:2621–30. 22679372 10.2147/IJN.S31757 PMC3368511

[CIT0010] Cuong NV, Jiang JL, Li YL, et al. (2010). Doxorubicin-loaded peg-pcl-peg micelle using xenograft model of nude mice: effect of multiple administration of micelle on the suppression of human breast cancer. Cancers (Basel) 3:61–78. 24212606 10.3390/cancers3010061 PMC3756349

[CIT0011] Dams T, Laverman P, Oyen WG, et al. (2000). Accelerated blood clearance and altered biodistribution of repeated injections of sterically stabilized liposomes. J Pharmacol Exp Ther 292:1071–9. 10688625

[CIT0012] Danquah MK, Zhang XA, Mahato RI. (2011). Extravasation of polymeric nanomedicines across tumor vasculature. Adv Drug Deliv Rev 63:623–39. 21144874 10.1016/j.addr.2010.11.005

[CIT0014] Dong D, Xiang B, Gao W, et al. (2013). pH-responsive complexes using prefunctionalized polymers for synchronous delivery of doxorubicin and siRNA to cancer cells. Biomaterials 34:4849–59. 23541420 10.1016/j.biomaterials.2013.03.018

[CIT0015] Dong H, Tang M, Li Y, et al. (2015). Disulfide-bridged cleavable PEGylation in polymeric nanomedicine for controlled therapeutic delivery. Nanomedicine 10:1941–58. 26139127 10.2217/nnm.15.38

[CIT0016] Du Y, Yang D, Sun S, et al. (2015). Preparation of pH-stimuli-responsive PEG-TGA/TGH-capped CdTe QDs and their application in cell labeling. Luminescence 30:519–25. 25244429 10.1002/bio.2770

[CIT0017] Etrych T, Kovar L, Strohalm J, et al. (2011). Biodegradable star HPMA polymer-drug conjugates: Biodegradability, distribution and anti-tumor efficacy. J Control Release 154:241–8. 21699933 10.1016/j.jconrel.2011.06.015

[CIT0018] Felber AE, Dufresne MH, Leroux JC. (2012). pH-sensitive vesicles, polymeric micelles, and nanospheres prepared with polycarboxylates. Adv Drug Deliv Rev 64:979–92. 21996056 10.1016/j.addr.2011.09.006

[CIT0019] Fretz MM, Koning GA, Mastrobattista E, et al. (2004). OVCAR-3 cells internalize TAT-peptide modified liposomes by endocytosis. Biochim Biophys Acta 1665:48–56. 15471570 10.1016/j.bbamem.2004.06.022

[CIT0020] Ghigo G, Maranzana A, Causà M, Tonachini G. (2006). Theoretical mechanistic studies on oxidation reactions of some saturated and unsaturated organic molecules. Theoretical Chem Accounts 117:699–707.

[CIT0021] Hammer N, Brandl FP, Kirchhof S, Goepferich AM. (2014). Cleavable carbamate linkers for controlled protein delivery from hydrogels. J Control Release 183:67–76. 24680687 10.1016/j.jconrel.2014.03.031

[CIT0022] Hansen CB, Kao GY, Moase EH, et al. (1995). Attachment of antibodies to sterically stabilized liposomes: evaluation, comparison and optimization of coupling procedures. Biochim Biophys Acta 1239:133–44. 7488618 10.1016/0005-2736(95)00138-s

[CIT0023] Hatakeyama H, Akita H, Kogure K, et al. (2007). Development of a novel systemic gene delivery system for cancer therapy with a tumor-specific cleavable PEG-lipid. Gene Ther 14:68–77. 16915290 10.1038/sj.gt.3302843

[CIT0024] Hatakeyama H, Akita H, Ito E, et al. (2011). Systemic delivery of siRNA to tumors using a lipid nanoparticle containing a tumor-specific cleavable PEG-lipid. Biomaterials 32:4306–16. 21429576 10.1016/j.biomaterials.2011.02.045

[CIT0025] Hatakeyama H, Ito E, Akita H, et al. (2009). A pH-sensitive fusogenic peptide facilitates endosomal escape and greatly enhances the gene silencing of siRNA-containing nanoparticles in vitro and in vivo. J Control Release 139:127–32. 19540888 10.1016/j.jconrel.2009.06.008

[CIT0026] Hatakeyama H, Akita H, Harashima H. (2013). The polyethyleneglycol dilemma: advantage and disadvantage of PEGylation of liposomes for systemic genes and nucleic acids delivery to tumors. Biol Pharm Bull 36:892–9. 23727912 10.1248/bpb.b13-00059

[CIT0027] Haupenthal J, Baehr C, Kiermayer S, et al. (2006). Inhibition of RNAse A family enzymes prevents degradation and loss of silencing activity of siRNAs in serum. Biochem Pharmacol 71:702–10. 16376306 10.1016/j.bcp.2005.11.015

[CIT0028] He ZY, Chu BY, Wei XW, et al. (2014). Recent development of poly(ethylene glycol)-cholesterol conjugates as drug delivery systems. Int J Pharm 469:168–78. 24768727 10.1016/j.ijpharm.2014.04.056

[CIT0029] Hu F, Liu L, Du Y, Yuan H. (2009). Synthesis and antitumor activity of doxorubicin conjugated stearic acid-g-chitosan oligosaccharide polymeric micelles. Biomaterials 30:6955–63. 19782395 10.1016/j.biomaterials.2009.09.008

[CIT0030] Ishida T, Kirchmeier MJ, Moase EH, et al. (2001). Targeted delivery and triggered release of liposomal doxorubicin enhances cytotoxicity against human B lymphoma cells. Biochimica Et Biophysica Acta 1515:144–58. 11718670 10.1016/s0005-2736(01)00409-6

[CIT0031] Ishihara T, Maeda T, Sakamoto H, et al. (2010). Evasion of the accelerated blood clearance phenomenon by coating of nanoparticles with various hydrophilic polymers. Biomacromolecules 11:2700–6. 20795699 10.1021/bm100754e

[CIT0032] Jiang T, Li YM, Lv Y, et al. (2013). Amphiphilic polycarbonate conjugates of doxorubicin with pH-sensitive hydrazone linker for controlled release. Colloids Surf B Biointerfaces 111:542–8. 23893028 10.1016/j.colsurfb.2013.06.054

[CIT0033] Kale AA, Torchilin VP. (2007a). Design, synthesis, and characterization of pH-sensitive PEG-PE conjugates for stimuli-sensitive pharmaceutical nanocarriers: the effect of substitutes at the hydrazone linkage on the ph stability of PEG-PE conjugates. Bioconjug Chem 18:363–70. 17309227 10.1021/bc060228x PMC2538438

[CIT0034] Kale AA, Torchilin VP. (2007b). “Smart” drug carriers: PEGylated TATp-modified pH-sensitive liposomes. J Liposome Res 17:197–203. 18027240 10.1080/08982100701525035 PMC3432921

[CIT0035] Kelly GJ, Kia AF, Hassan F, et al. (2016). Polymeric prodrug combination to exploit the therapeutic potential of antimicrobial peptides against cancer cells. Org Biomol Chem 14:9278–86. 27722734 10.1039/c6ob01815g

[CIT0036] Kierstead PH, Okochi H, Venditto VJ, et al. (2015). The effect of polymer backbone chemistry on the induction of the accelerated blood clearance in polymer modified liposomes. J Control Release 213:1–9. 26093095 10.1016/j.jconrel.2015.06.023 PMC4684485

[CIT0037] Kim JH, Kim TH, Jang JW, et al. (2001). Analysis of matrix metalloproteinase mRNAs expressed inhepatocellular carcinoma cell lines. Mol Cells 12:32–40. 11561728

[CIT0038] Kim JY, Kim S, Pinal R, Park K. (2011). Hydrotropic polymer micelles as versatile vehicles for delivery of poorly water-soluble drugs. J Control Release 152:13–20. 21352878 10.1016/j.jconrel.2011.02.014

[CIT0039] Komeda C, Ikeda A, Kikuchi J, et al. (2013). A photo-triggerable drug carrier based on cleavage of PEG lipids by photosensitiser-generated reactive singlet oxygen. Org Biomol Chem 11:2567–70. 23307046 10.1039/c2ob27199k

[CIT0040] Koren E, Apte A, Jani A, Torchilin VP. (2012). Multifunctional PEGylated 2C5-immunoliposomes containing pH-sensitive bonds and TAT peptide for enhanced tumor cell internalization and cytotoxicity. J Control Release 160:264–73. 22182771 10.1016/j.jconrel.2011.12.002 PMC3361627

[CIT0041] Koutroumanis KP, Holdich RG, Georgiadou S. (2013). Synthesis and micellization of a pH-sensitive diblock copolymer for drug delivery. Int J Pharm 455:5–13. 23850624 10.1016/j.ijpharm.2013.06.071

[CIT0042] Kuai R, Yuan W, Li W, et al. (2011). Targeted delivery of cargoes into a murine solid tumor by a cell-penetrating peptide and cleavable poly(ethylene glycol) comodified liposomal delivery system via systemic administration. Mol Pharmaceutics 8:2151–61. 10.1021/mp200100f 21981683

[CIT0043] Kuai R, Yuan W, Qin Y, et al. (2010). Efficient delivery of payload into tumor cells in a controlled manner by TAT and thiolytic cleavable PEG co-modified liposomes. Mol Pharmaceutics 7:1816–26. 10.1021/mp100171c 20701288

[CIT0044] Kulkarni PS, Haldar MK, Nahire RR, et al. (2014). Mmp-9 responsive PEG cleavable nanovesicles for efficient delivery of chemotherapeutics to pancreatic cancer. Mol Pharmaceutics 11:2390–9. 10.1021/mp500108p PMC4096225 24827725

[CIT0045] Lankveld DP, Rayavarapu RG, Krystek P, et al. (2011). Blood clearance and tissue distribution of PEGylated and non-PEGylated gold nanorods after intravenous administration in rats. Nanomedicine (Lond) 6:339–49. 21385136 10.2217/nnm.10.122

[CIT0046] Laverman P, Carstens MG, Boerman OC, et al. (2001). Factors affecting the accelerated blood clearance of polyethylene glycol-liposomes upon repeated injection. J Pharmacol Exp Ther 298:607–12. 11454922

[CIT0047] Lee ES, Na K, Bae YH. (2005). Super pH-sensitive multifunctional polymeric micelle. Nano Lett 5:325–9. 15794620 10.1021/nl0479987

[CIT0048] Li C, Cao J, Wang Y, et al. (2012a). Accelerated blood clearance of pegylated liposomal topotecan: influence of polyethylene glycol grafting density and animal species. J Pharm Sci 101:3864–76. 22777607 10.1002/jps.23254

[CIT0049] Li Y, Liu J, Liu B, Tomczak N. (2012b). Highly emissive PEG-encapsulated conjugated polymer nanoparticles. Nanoscale 4:5694–702. 22878417 10.1039/c2nr31267k

[CIT0050] Lila AS, Kiwada H, Ishida T. (2013). The accelerated blood clearance (ABC) phenomenon: Clinical challenge and approaches to manage. J Control Release 172:38–47. 23933235 10.1016/j.jconrel.2013.07.026

[CIT0051] Lin J, Li Y, Li Y, et al. (2015). Drug/dye-loaded, multifunctional peg-chitosan-iron oxide nanocomposites for methotraxate synergistically self-targeted cancer therapy and dual model imaging. ACS Appl Mater Interfaces 7:11908–20. 25978458 10.1021/acsami.5b01685

[CIT0052] Maeda N, Takeuchi Y, Takada M, et al. (2004). Anti-neovascular therapy by use of tumor neovasculature-targeted long-circulating liposome. J Control Release 100:41–52. 15491809 10.1016/j.jconrel.2004.07.033

[CIT0053] Maeda T, Fujimoto K. (2006). A reduction-triggered delivery by a liposomal carrier possessing membrane-permeable ligands and a detachable coating. Colloids Surf B Biointerfaces 49:15–21. 16574385 10.1016/j.colsurfb.2006.02.006

[CIT0054] Magarkar A, Karakas E, Stepniewski M, et al. (2012). Molecular dynamics simulation of PEGylated bilayer interacting with salt ions: a model of the liposome surface in the bloodstream. J Phys Chem B 116:4212–9. 22420691 10.1021/jp300184z

[CIT0055] Mei L, Fu L, Shi K, et al. (2014). Increased tumor targeted delivery using a multistage liposome system functionalized with RGD, TAT and cleavable PEG. Int J Pharm 468:26–38. 24709209 10.1016/j.ijpharm.2014.04.008

[CIT0056] Mercadal M, Domingo JC, Petriz J, et al. (2000). Preparation of immunoliposomes bearing poly(ethylene glycol)-coupled monoclonal antibody linked via a cleavable disulfide bond for ex vivo applications. Biochim Biophys Acta 1509:299–310. 11118541 10.1016/s0005-2736(00)00305-9

[CIT0057] Mishra S, Webster P, Davis ME. (2004). PEGylation significantly affects cellular uptake and intracellular trafficking of non-viral gene delivery particles. Eur J Cell Biol 83:97–111. 15202568 10.1078/0171-9335-00363

[CIT0058] Oberoi HS, Yorgensen YM, Morasse A, et al. (2016). PEG modified liposomes containing CRX-601 adjuvant in combination with methylglycol chitosan enhance the murine sublingual immune response to influenza vaccination. J Control Release 223:64–74. 26551346 10.1016/j.jconrel.2015.11.006 PMC4729458

[CIT0059] Oumzil K, Khiati S, Grinstaff MW, Barthelemy P. (2011). Reduction-triggered delivery using nucleoside-lipid based carriers possessing a cleavable PEG coating. J Control Release 151:123–30. 21354443 10.1016/j.jconrel.2011.02.008

[CIT0060] Paliwal SR, Paliwal R, Vyas SP. (2015). A review of mechanistic insight and application of pH-sensitive liposomes in drug delivery. Drug Deliv 22:231–42. 24524308 10.3109/10717544.2014.882469

[CIT0061] Pappalardo JS, Quattrocchi V, Langellotti C, et al. (2009). Improved transfection of spleen-derived antigen-presenting cells in culture using TATp-liposomes. J Control Release 134:41–6. 19059290 10.1016/j.jconrel.2008.11.006

[CIT0063] Patil R, Portilla-Arias J, Ding H, et al. (2012). Cellular delivery of doxorubicin via pH-controlled hydrazone linkage using multifunctional nano vehicle based on poly(β-l-malic acid) ). Int J Mol Sci 13:11681–93. 23109877 10.3390/ijms130911681 PMC3472769

[CIT0064] Prabaharan M, Grailer JJ, Pilla S, et al. (2009). Amphiphilic multi-arm-block copolymer conjugated with doxorubicin via pH-sensitive hydrazone bond for tumor-targeted drug delivery. Biomaterials 30:5757–66. 19643472 10.1016/j.biomaterials.2009.07.020

[CIT0065] Quader S, Cabral H, Mochida Y, et al. (2014). Selective intracellular delivery of proteasome inhibitors through pH-sensitive polymeric micelles directed to efficient antitumor therapy. J Control Release 188:67–77. 24892974 10.1016/j.jconrel.2014.05.048

[CIT0066] Rodrigues PC, Beyer U, Schumacher P, et al. (1999). Acid-sensitive polyethylene glycol conjugates of doxorubicin: preparation, in vitro efficacy and intracellular distribution. Bioorg Med Chem 7:2517–24. 10632061 10.1016/s0968-0896(99)00209-6

[CIT0067] Romberg B, Hennink WE, Storm G. (2008). Sheddable coatings for long-circulating nanoparticles. Pharm Res 25:55–71. 17551809 10.1007/s11095-007-9348-7 PMC2190344

[CIT0068] Saadati R, Dadashzadeh S, Abbasian Z, Soleimanjahi H. (2013). Accelerated blood clearance of PEGylated PLGA nanoparticles following repeated injections: effects of polymer dose, PEG coating, and encapsulated anticancer drug. Pharm Res 30:985–95. 23184228 10.1007/s11095-012-0934-y

[CIT0069] Sanchez L, Yi Y, Yu Y. (2017). Effect of partial PEGylation on particle uptake by macrophages. Nanoscale 9:288–97. 27909711 10.1039/c6nr07353k PMC6397647

[CIT0070] Sawant RM, Hurley JP, Salmaso S, et al. (2006). "SMART” drug delivery systems: double-targeted pH-responsive pharmaceutical nanocarriers. Bioconjugate Chem 17:943–9. 10.1021/bc060080h PMC2538444 16848401

[CIT0071] Sawant RP, Sriraman SK, Navarro G, et al. (2012). Polyethyleneimine-lipid conjugate-based pH-sensitive micellar carrier for gene delivery. Biomaterials 33:3942–51. 22365809 10.1016/j.biomaterials.2011.11.088 PMC3527089

[CIT0072] Shaunak S, Godwin A, Choi JW, et al. (2006). Site-specific PEGylation of native disulfide bonds in therapeutic proteins. Nat Chem Biol 2:312–3. 16633351 10.1038/nchembio786

[CIT0073] Shete HK, Prabhu RH, Patravale VB. (2014). Endosomal escape: a bottleneck in intracellular delivery. J Nanosci Nanotechnol 14:460–74. 24730275 10.1166/jnn.2014.9082

[CIT0074] Shimizu T, Mima Y, Hashimoto Y, et al. (2015). Anti-PEG IgM and complement system are required for the association of second doses of PEGylated liposomes with splenic marginal zone B cells. Immunobiology 220:1151–60. 26095176 10.1016/j.imbio.2015.06.005

[CIT0075] Shin J, Shum P, Thompson D. (2003). Acid-triggered release via dePEGylation of DOPE liposomes containing acid-labile vinyl ether PEG-lipids. J Control Release 91:187–200. 12932651 10.1016/s0168-3659(03)00232-3

[CIT0077] Song LY, Ahkong QF, Rong Q, et al. (2002). Characterization of the inhibitory effect of PEG-lipid conjugates on the intracellular delivery of plasmid and antisense DNA mediated by cationic lipid liposomes. Biochimica Et Biophysica Acta 1558:1–13. 11750259 10.1016/s0005-2736(01)00399-6

[CIT0078] Sun CY, Liu Y, Du JZ, et al. (2016). Facile generation of tumor-ph-labile linkage-bridged block copolymers for chemotherapeutic delivery. Angew Chem Int Ed Engl 55:1010–4. 26756443 10.1002/anie.201509507

[CIT0079] Tagalakis AD, Kenny GD, Bienemann AS, et al. (2014). PEGylation improves the receptor-mediated transfection efficiency of peptide-targeted, self-assembling, anionic nanocomplexes. J Control Release 174:177–87. 24269968 10.1016/j.jconrel.2013.11.014

[CIT0080] Tang J, Fu H, Kuang Q, et al. (2014). Liposomes co-modified with cholesterol anchored cleavable PEG and octaarginines for tumor targeted drug delivery. J Drug Target 22:313–26. 24404866 10.3109/1061186X.2013.875029

[CIT0081] Terada T, Iwai M, Kawakami S, et al. (2006). Novel PEG-matrix metalloproteinase-2 cleavable peptide-lipid containing galactosylated liposomes for hepatocellular carcinoma-selective targeting. J Control Release 111:333–42. 16488046 10.1016/j.jconrel.2005.12.023

[CIT0082] Torchilin VP. (2007). Targeted pharmaceutical nanocarriers for cancer therapy and imaging. Aaps J 9:E128–47. 17614355 10.1208/aapsj0902015 PMC2751402

[CIT0083] Torchilin VP. (2008). Tat peptide-mediated intracellular delivery of pharmaceutical nanocarriers. Adv Drug Deliv Rev 60:548–58. 18053612 10.1016/j.addr.2007.10.008

[CIT0084] Torchilin VP, Levchenko TS, Rammohan R, et al. (2003). Cell transfection *in vitro* and *in vivo* with nontoxic TAT peptide-liposome-DNA complexes. Proc Natl Acad Sci USA 100:1972–7. 12571356 10.1073/pnas.0435906100 PMC149943

[CIT0085] Torchilin VP, Rammohan R, Weissig V, Levchenko TS. (2001). TAT peptide on the surface of liposomes affords their efficient intracellular delivery even at low temperature and in the presence of metabolic inhibitors. Proc Natl Acad Sci USA 98:8786–91. 11438707 10.1073/pnas.151247498 PMC37513

[CIT0086] Vance ME, Marr LC. (2015). Exposure to airborne engineered nanoparticles in the indoor environment. Atmos Environ 106:503–9.

[CIT0087] Vandenbroucke RE, De Smedt SC, Demeester J, Sanders NN. (2007). Cellular entry pathway and gene transfer capacity of TAT-modified lipoplexes. Biochim Biophys Acta 1768:571–9. 17188643 10.1016/j.bbamem.2006.11.006

[CIT0088] Wang C, Cheng X, Su Y, et al. (2015). Accelerated blood clearance phenomenon upon cross-administration of PEGylated nanocarriers in beagle dogs. Int J Nanomedicine 10:3533–45. 25999716 10.2147/IJN.S82481 PMC4437610

[CIT0089] Wang H, Su W, Wang S, et al. (2012). Smart multifunctional core-shell nanospheres with drug and gene co-loaded for enhancing the therapeutic effect in a rat intracranial tumor model. Nanoscale 4:6501–8. 22961067 10.1039/c2nr31263h

[CIT0090] Wang H, Zhao P, Liang X, et al. (2010a). Folate-PEG coated cationic modified chitosan–cholesterol liposomes for tumor-targeted drug delivery. Biomaterials 31:4129–38. 20163853 10.1016/j.biomaterials.2010.01.089

[CIT0091] Wang L, Su Y, Wang X, et al. (2017). Effects of complement inhibition on the ABC phenomenon in rats. AJPS 12:250–8. 32104336 10.1016/j.ajps.2016.06.004 PMC7032085

[CIT0092] Wang M, Thanou M. (2010). Targeting nanoparticles to cancer. Pharmacol Res 62:90–9. 20380880 10.1016/j.phrs.2010.03.005

[CIT0093] Wang S, Wang H, Liu Z, et al. (2014). Smart pH- and reduction-dual-responsive folate-PEG-coated polymeric lipid vesicles for tumor-triggered targeted drug delivery. Nanoscale 6:7635–42. 24898341 10.1039/c4nr00843j

[CIT0094] Wang S, Xu H, Xu J, et al. (2010b). Sustained liver targeting and improved antiproliferative effect of doxorubicin liposomes modified with galactosylated lipid and PEG-lipid. AAPS PharmSciTech 11:870–7. 20490957 10.1208/s12249-010-9450-8 PMC2902302

[CIT0095] Wei M, Xu Y, Zou Q, et al. (2012). Hepatocellular carcinoma targeting effect of PEGylated liposomes modified with lactoferrin. Eur J Pharm Sci 46:131–41. 22369856 10.1016/j.ejps.2012.02.007

[CIT0096] Wu X, Yan G. (2015). Copper nanopowder catalyzed cross-coupling of diaryl disulfides with aryl iodides in PEG-400. Synlett 26:537–42.

[CIT0097] Wu Y, Chen W, Meng F, et al. (2012). Core-crosslinked pH-sensitive degradable micelles: a promising approach to resolve the extracellular stability versus intracellular drug release dilemma. J Control Release 164:338–45. 22800578 10.1016/j.jconrel.2012.07.011

[CIT0098] Xu H, Deng Y, Chen D, et al. (2008). Esterase-catalyzed dePEGylation of pH-sensitive vesicles modified with cleavable PEG-lipid derivatives. J Control Release 130:238–45. 18657874 10.1016/j.jconrel.2008.05.009

[CIT0099] Xu H, Ye F, Hu M, et al. (2014). Influence of phospholipid types and animal models on the accelerated blood clearance phenomenon of PEGylated liposomes upon repeated injection. Drug Delivery 22:598–607. 24524364 10.3109/10717544.2014.885998

[CIT0100] Xu H, Wang K, Deng Y, Chen D. (2010). Effects of cleavable PEG-cholesterol derivatives on the accelerated blood clearance of PEGylated liposomes. Biomaterials 31:4757–63. 20303164 10.1016/j.biomaterials.2010.02.049

[CIT0101] Yan X, Yang X, Tong X, Huang Y. (2014). A method to accelerate the gelation of disulfide-crosslinked hydrogels. Chin J Polym Sci 33:118–27.

[CIT0102] Yoshino K, Taguchi K, Mochizuki M, et al. (2012). Novel analytical method to evaluate the surface condition of polyethylene glycol-modified liposomes. Colloids and Surfaces A Physicochem Eng Aspects 397:73–9.

[CIT0103] Yuan H, Chen CY, Chai GH, et al. (2013). Improved transport and absorption through gastrointestinal tract by PEGylated solid lipid nanoparticles. Mol Pharm 10:1865–73. 23495754 10.1021/mp300649z

[CIT0104] Zeng S, Wu F, Li B, et al. (2014). Synthesis, characterization, and evaluation of a novel amphiphilic polymer RGD-PEG-Chol for target drug delivery system. Scientific World J 2014:546176. 10.1155/2014/546176 PMC3918714 24578646

[CIT0105] Zhang F, Lin J, Zhao G. (2016). Preparation and characterization of modified soda lignin with polyethylene glycol. Materials(Basel) 9:1–9. 10.3390/ma9100822 PMC5456608 28773943

[CIT0106] Zhang J, Zalipskyb S, Mullah N, et al. (2004). Pharmaco attributes of dioleoylphosphatidylethanolamine/cholesterylhemisuccinate liposomes containing different types of cleavable lipopolymers. Pharmacol Res 49:185–98. 14643699 10.1016/j.phrs.2003.09.003

[CIT0107] Zhang Y, Mintzer E, Uhrich KE. (2016). Synthesis and characterization of PEGylated bolaamphiphiles with enhanced retention in liposomes. J Colloid Interface Sci 482:19–26. 27485501 10.1016/j.jcis.2016.07.013

[CIT0108] Zhao G, Long L, Zhang L, et al. (2017). Smart pH-sensitive nanoassemblies with cleavable PEGylation for tumor targeted drug delivery. Sci Rep 7:3383–93. 28611459 10.1038/s41598-017-03111-2 PMC5469818

[CIT0109] Zheng J, Wan Y, Elhissi A, et al. (2014). Targeted paclitaxel delivery to tumors using cleavable PEG-conjugated solid lipid nanoparticles. Pharm Res 31:2220–33. 24595496 10.1007/s11095-014-1320-8

[CIT0110] Zhou L, Cheng R, Tao H, et al. (2011). Endosomal pH-activatable poly(ethylene oxide)-graft-doxorubicin prodrugs: synthesis, drug release, and biodistribution in tumor-bearing mice. Biomacromolecules 12:1460–7. 21332185 10.1021/bm101340u

[CIT0111] Zhou L, Liang D, He X, et al. (2012). The degradation and biocompatibility of pH-sensitive biodegradable polyurethanes for intracellular multifunctional antitumor drug delivery. Biomaterials 33:2734–45. 22236829 10.1016/j.biomaterials.2011.11.009

[CIT0112] Zhu H, Dong C, Dong H, et al. (2014). Cleavable PEGylation and hydrophobic histidylation of polylysine for siRNA delivery and tumor gene therapy. ACS Appl Mater Interfaces 6:10393–407. 24892498 10.1021/am501928p

